# Effects of computerized cognitive training on biomarker responses in older adults with mild cognitive impairment: A scoping review

**DOI:** 10.1002/hsr2.2175

**Published:** 2024-06-17

**Authors:** Hiroshi Hayashi, Toshimasa Sone, Kazuaki Iokawa, Koshi Sumigawa, Takaaki Fujita, Hironori Kawamata, Akihiko Asao, Iori Kawasaki, Maki Ogasawara, Shinobu Kawakatsu

**Affiliations:** ^1^ Department of Occupational Therapy Fukushima Medical University School of Health Sciences Fukushima Japan; ^2^ Department of Neuropsychiatry, Aizu Medical Center Fukushima Medical University Aizuwakamatsu Japan

**Keywords:** biomarker, brain‐derived neurotrophic factor, computerized cognitive training, electroencephalography, functional near‐infrared spectroscopy, magnetic resonance imaging, mild cognitive impairment

## Abstract

**Background and Aims:**

Mild cognitive impairment (MCI) is a widespread condition in older individuals, posing significant risk of dementia. However, limited research has been conducted to explore effective interventions and clarify their impact at the neural level. Therefore, this study aimed to investigate the effects of computerized cognitive training (CCT) and explore the associated neural mechanisms in preventing dementia in older individuals with MCI, with a view to inform future intervention efforts.

**Methods:**

We reviewed the effects of CCT on biomarker outcomes in older adults with MCI. The search was conducted for studies published between 2010 and May 10, 2023, using three search engines: PubMed, Scopus, and Cumulative Index to Nursing and Allied Health Literature. The inclusion criteria were as follows: studies that involved participants diagnosed with MCI, included CCT, included quantitative assessment of biomarker results, and conducted randomized controlled trials.

**Results:**

Sixteen studies that used biomarkers, including magnetic resonance imaging, electroencephalography (EEG), functional near‐infrared spectroscopy (fNIRS), and blood or salivary biomarkers, were extracted. The results showed that CCT caused changes in structure and function within the main brain network, including the default mode network, and decreased both theta rhythm activity on EEG and prefrontal activity on fNIRS, with improvement in cognitive function. Furthermore, CCT combined with physical exercise showed more significant structural and functional changes in extensive brain regions compared with CCT alone. Virtual reality‐based cognitive training improved not only executive function but also instrumental activities of daily living.

**Conclusion:**

CCT causes functional and structural changes in extensive brain regions and improves cognitive function in older adults with MCI. Our findings highlight the potential of individualized intervention methods and biomarker assessment according to the specific causes of MCI. Future research should aim to optimize these personalized therapeutic strategies to maximize the benefits of CCT in older adults with MCI.

## Key points

- Computerized cognitive training (CCT) causes functional and structural changes in extensive brain regions in older adults with mild cognitive impairment (MCI), with improvement in cognitive function.
- The effect of CCT combined with physical exercise may be stronger and longer‐lasting than that of CCT alone.
- Virtual reality‐based cognitive training may improve not only executive function but also instrumental activities of daily living.

## 1 INTRODUCTION

Mild cognitive impairment (MCI) is characterized by a decline in cognitive function, but it typically preserves intellectual and daily life skills in older individuals.^1^ The incidence rate of MCI ranges from 15% to 20% in older adults aged ≥65 years,^2^ with Alzheimer's disease (AD) being the most common cause of MCI.^1^ MCI is considered a prodromal stage of dementia, with the condition progressing to dementia in 4%–22% of cases and reverting to normal cognition in 20%–57% of cases over 4 years.^3^ Early interventions at the MCI stage are important for preventing dementia; however, no pharmacotherapy has been established for preventing progression to dementia.

Nonpharmacological interventions, including cognitive training, physical exercise, and social participation, are effective in delaying cognitive decline in older adults with MCI.^4^, ^5^, ^6^, ^7^, ^8^ Although these interventions are recommended to prevent dementia in older individuals with MCI, it is difficult for these individuals to continue training after intervention cessation.^7^ Hence, intervention methods that allow participants to continue training without difficulty are required.

With rapid advancements in computer science over the past decade, computerized cognitive training (CCT) using virtual reality (VR) and information and communication technology (ICT) has been increasingly used to prevent dementia in older individuals with MCI.^9^, ^10^ Furthermore, mandatory social distancing due to the coronavirus disease pandemic has facilitated CCT using ICT.^11^ CCT is structured, and previous studies have reported the use of the Cognitive Operations Gear COG Pack (COGPACK),^12^ Cogmed Working Memory Training Program,^13^ and Comprehensive and Complex Cognitive Stimulation Program.^14^ CCT has been considered a flexible and convenient method to delay cognitive decline, as CCT programs have reportedly improved cognitive function in older individuals with MCI,^15^, ^16^ and the effects of CCT are comparable to those of conventional cognitive training.^17^ Moreover, CCT includes games that use commercially available game software, such as the Xbox 360 Kinect and VR technology, which provide sensory feedback through auditory, visual, and tactile stimulation.^18^ These training programs allow participants to continue the training safely and enjoyably.^19^ Furthermore, the combination of CCT and physical exercise is more effective than training alone and is increasingly used to prevent dementia.^20^, ^21^, ^22^, ^23^, ^24^

Nevertheless, although several research groups have demonstrated improvements in cognitive function due to CCT, only a few studies have examined the neural mechanism of the effects of CCT on cognitive function in older adults with MCI. Recently, the number of studies using biomarkers to estimate the effects of CCT in older adults with MCI has gradually increased, and the following effects have been reported: elevation of serum brain‐derived neurotrophic factor (BDNF) level^25^; recovery of increased delta and theta bands on electroencephalography (EEG)^21^; increased gray matter volume,^26^ cerebral blood flow, or brain metabolism^27^, ^28^; and enhanced functional connectivity (FC) in neural networks.^29^ Moreover, the effect on biomarkers after intervention differs depending on CCT content and the presence or absence of a combination of physical exercises.^12^, ^17^, ^25^, ^29^

Although a previous review demonstrated the effects of CCT on neuroimaging outcomes in older adults in 2017, it included healthy older adults and limited biomarkers for neuroimaging.^30^ To the best of our knowledge, no review has focused on biomarkers for investigating the effects of CCT in older adults with MCI. Therefore, this study aimed to review the findings of biomarker assessments for evaluating the effects of CCT, to understand the underlying neural mechanism of CCT in preventing dementia in older individuals with MCI, and to provide directions for future intervention efforts.

## 2 MATERIALS AND METHODS

### 2.1 Study design and participants

We performed a peer scoping review according to the Preferred Reporting Items for Systematic Reviews and Meta‐Analyses for Scoping Reviews guidelines.^31^

The inclusion criteria were as follows: studies (1) assessing participants diagnosed with or screened for MCI; (2) evaluating computerized training involving all cognitive training using computer technology, such as VR, ICT, and artificial intelligence; (3) quantitatively assessing biomarkers; and (4) conducting a randomized controlled trial (RCT). The exclusion criteria were as follows: studies (1) assessing MCI due to psychiatric disease, cerebrovascular disease, multiple sclerosis, Parkinson's disease, brain injury, encephalitis, epilepsy, human immunodeficiency virus infection, and other physical illnesses, including heart failure and malignancy; (2) evaluating the combination of CCT and other interventions, except physical exercise (e.g., pharmacotherapy, diet therapy, or transcranial alternating current stimulation); (3) published in languages other than English; and (4) with abstracts only.

### 2.2 Literature search process

The search was conducted for studies published between 2010 and May 10, 2023, as there were no studies examining changes in biomarkers after CCT before 2010. Three search engines were used: PubMed, Scopus, and Cumulative Index to Nursing and Allied Health Literature. The following keywords were used: (“dementia” or “Alzheimer” or “cognitive impairment”) and (“training” or “stimulation” or “rehabilitation” or “intervention” or “trial”) and (“biomarker” or “Near‐infrared spectroscopy” or “Magnetic Resonance Imaging” or “Single Photon Emission Computed Tomography” or “Positron Emission Tomography” or “electroencephalography” or “saliva” or “blood” or “serum” or “plasma” or “cerebrospinal fluid”) and (“digital” or “virtual reality” or “computerized” or “computer” or “exergame” or “robot” or “telemedicine” or “Artificial Intelligence” or “Information and Communication Technology”). Two researchers (HH and ST) independently conducted the initial search of the databases by reading all the abstracts of the articles generated to confirm that they targeted computerized training and biomarkers in older adults with MCI. The entire texts of the extracted studies were then reviewed against the inclusion criteria. The researchers held discussions when they disagreed with their respective judgments on the criteria. We also used snowball sampling for the reference lists of relevant articles. Study citations were imported into a reference management software (EndNote 20, Clarivate Analytics) for selection.

## 3 RESULTS

### 3.1 Literature search findings

**Figure 1:**
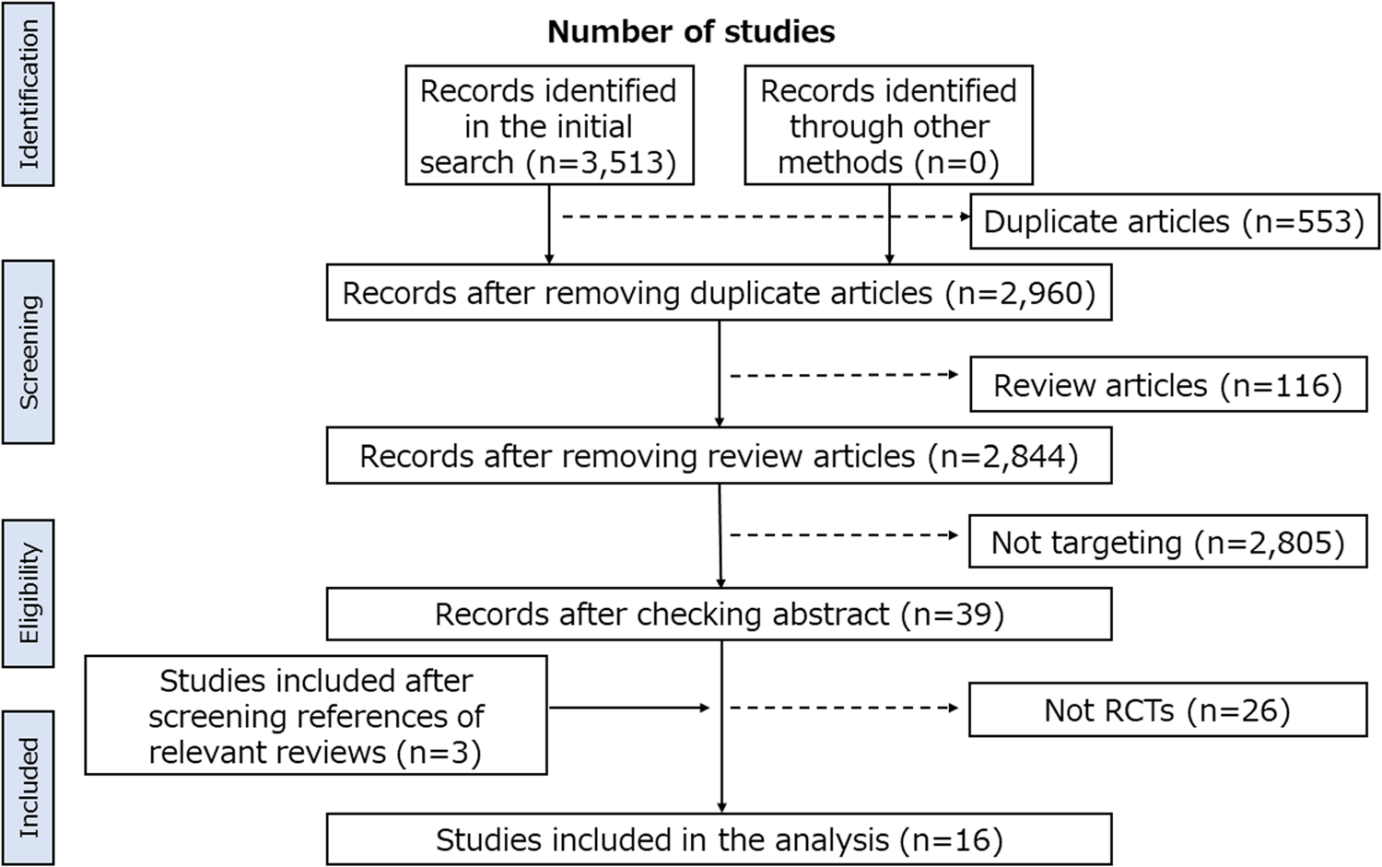
Study flowchart. RCT, randomized controlled trial.

Based on a search of the three databases, 3513 studies were identified in the initial search, of which 553 duplicates and 116 reviews were removed. Of the three search engines used, the initial search results in PubMed are shown in supplementary material 1. Articles that did not target interventions for MCI, computerized training, or assessment of biomarkers (*n* = 2805) were excluded. The entire text of 39 articles was read by two researchers, and 13 articles that met the inclusion criteria were selected. Additionally, three studies were included after screening the references of relevant reviews. Finally, 16 studies were selected for analysis (Figure 1). The characteristics of the studies are presented in Table 1.

**Table 1:** Details of randomized controlled trials on the effects of computerized cognitive training on biomarker responses in older adults with mild cognitive impairment.

Authors (year)	Type of intervention	Sample size	Length per session, frequency, duration	Biomarker outcome measures	Cognition measured test	Cognition outcome	Biomarker outcome
Computerized cognitive training program including virtual reality							
Yang et al. (2022)^32^	Virtual reality‐based cognitive training (VRCT) Exercise Control: education seminars	VRCT group: 33 Exercise group: 33 Control group: 33	VRCT program: 100 min, 3 times/week for 8 weeks Exercise program: 60 min, 2 times/week for 12 weeks Control group: 30 min, once a week for 8 weeks	EEG	MMSE TMT‐A SDMT	Results showed a significant improvement of MMSE scores in both the VRCT and exercise groups (*p* < 0.05).	EEG showed a significant decrease in theta power in the parietal regions during follow up in the VRCT group compared with that in the exercise group (*p* < 0.05), and a significant decrease in the theta/beta ratio compared with that in the control group in both intervention groups (*p* < 0.05).
Kang et al. (2021)^9^	Virtual reality‐assisted cognitive training (VRT) Control: usual care	VRT group: 23 Control group: 18	VRT program: 20–30 min, twice/week for 4 weeks	Functional MRI	RCFT MMSE TMT‐A, B BNT SVLT SCWT	The VRT group showed a significant improvement in the RCFT copy task compared to the control group (*p* = 0.001).	Functional connectivity in the visual network related to improvements in the RCFT copy task was significantly enhanced in the VRT group compared to that in the control group (*p* < 0.05).
Thapa et al. (2020)^19^	Virtual reality‐based cognitive training (VRCT) Control: education seminars	VRCT group: 34 Control group: 33	VRCT program: 100 min, 3 times/week for 8 weeks Control group: 30–50 min, once a week for 8 weeks	EEG	MMSE TMT‐A, B SDMT	VR intervention group showed a significant improvement in executive function compared to control group (*p* = 0.03).	EEG showed a significant decrease in theta/beta ratio in the temporal (*p* = 0.035) and parietal (*p* = 0.027) regions in the VR intervention group. There were no significant changes in the control group.
Liao et al. (2020)^17^	Virtual reality‐based cognitive and physical training (VRT) Combined physical and cognitive training (CPCT)	VRT group: 21 CPCT group: 21	VRT program: 60 min (physical training for 40 min, cognitive training for 20 min), 3 times/week for 12 weeks CPCT program: 60 min, 3 times/week for 12 weeks	fNIRS	MoCA EXIT‐25 CVVLT	Both groups had improved executive function and verbal memory. Improvements in MoCA score (*p* < 0.001) and IADL (*p* < 0.001) were observed only in the VRT group.	Results showed decreases in the brain activation of the prefrontal areas after intervention in both groups (*p* < 0.05).
Computerized cognitive training program without virtual reality							
Luo et al. (2023)^33^	Remote Expressive Arts Program (rEAP) Health education (HE)	rEAP group: 38 HE group: 35	rEAP: 60 min, twice/week for 12 weeks HE: 30–60 min, twice/week for 12 weeks	Functional MRI	MoCA MMSE STT‐A, B VFT BNT SDMT	The rEAP group showed more significant improvements in MoCA‐J score (*p* = 0.012), memory (*p* = 0.036), and executive function (*p* = 0.009) than the HE group.	The activation in the right anterior cingulate cortex (*p* < 0.05) and left dorsolateral superior frontal gyrus (*p* < 0.05) significantly increased and decreased, respectively, in the rEAP and HE groups. In the rEAP group, the changes were significantly related to improved overall cognitive function (*p* < 0.05).
Petrella et al. (2023)^29^	Computerized cognitive games training (CCT) Web‐based crossword puzzles (CP)	CCT group: 39 CP group: 48	30‐min training sessions per week for 12 weeks. Subsequent booster training was composed of four 30‐min sessions, completed over 1 week and occurring at weeks 20, 32, 42, 52, 64, and 78.	Functional MRI	ADAS‐Cog	The CP group showed a more significant improvement in cognitive function than the CCT group (*p* = 0.04).^34^	There was no statistically significant treatment‐related difference in the change from baseline in within‐network connectivity. Within network functional connectivity (FC) in the default mode network (DMN) significantly decreased over the course of the trial in the games condition, but significantly increased in the crossword control group (*p* = 0.034). There was a significant decrease in between‐network FC involving the DMN and all other networks in the games group, whereas the crossword group showed significantly increased connectivity between these networks (*p* = 0.045).
Hol et al. (2022)^35^	Adaptive computerized working memory (WM) training (the tasks becomes more complex and difficult) Nonadaptive WM training (the participants trained at a fixed low level of difficulty)	Adaptive computerized WM training group: 32 Nonadaptive WM training group: 30	30–40 min, 5 times/week for 5 weeks	Structural MRI	WMS‐Ⅲ Digit Span backward WMS‐Ⅲ Spatial Spam backward WMS‐Ⅲ Letter‐Number Sequencing CVLT‐Ⅱ Trial B	No improvement in WM in both groups^13^	There were no significant structural cortical changes and no difference in cortical thickness after training in both groups. Cortical thickness in the prefrontal and paracentral regions increased significantly in Lim homeobox transcription factor‐alpha (LMX1A)‐AA carriers of the LMX1A polymorphism (*p* = 0.00043).
Quialheiro et al. (2022)^14^	Comprehensive and complex cognitive stimulation program (computer activity, physical activity, exchanging experiences in a conversation circle) Control subjects were instructed to perform memory exercise and were encouraged to participate in activities that provide new knowledge or skills.	Comprehensive and complex cognitive stimulation program: 32 Control group: 32	the session dosage was not reported 2 times/week for 12 weeks	Serum (BDNF, S100β, NSE)	MoCA MMSE	MoCA scores were significantly improved in the intervention group compared to those in the control group (*p* < 0.001).	BDNF level significantly decreased with no correlation with increased MoCA score in the intervention group (*p* < 0.001), while the changes in S100, calcium‐binding protein B (*p* = 0.603), and neuron‐specific enolase (*p* = 0.977) levels were not significant.
Park (2021)^36^	Cognitive‐exercise combined program Control: only executive function training	Cognitive‐exercise combined program group: 18 Control group: 18	40 min, twice/week for 8 weeks	fNIRS	TMT‐B	The experimental group achieved a significant improvement in the TMT‐B compared to the control group (*p* < 0.05).	The experimental group showed a significant decrease in activity in the prefrontal cortex during TMT‐B testing compared to the control group (*p* < 0.05).
Broadhouse et al. (2020)^37^	Computerized cognitive training using the COGPACK program (CCT)+ progressive resistance training (PRT) CCT+Sham PRT+Sham Control (Sham+Sham)	CCT + PRT group: 16 CCT+Sham group: 22 PRT+Sham group: 16 Control group: 24	12 months of no training and no supervision after cessation of four types of interventions as described by Suo et al. (2016).	Structural MRI Functional MRI	ADAS‐Cog	At 12 months after training cessation, there was no long‐term cognitive improvement in the CCT or the CCT + PRT group.	At 12 months after training cessation, The PRT + CCT group exhibited significantly slower atrophy rates in the left hippocampus compared to the control group (*p* = 0.042). fMRI showed significantly enhanced functional connectivity between the hippocampus and posterior cingulate in the CCT + PRT group (*p* = 0.018).
Amjad et al. (2019)^38^	Xbox 360 Kinect cognitive game training, Computerized cognitive training (CCT)+physical exercise Control: stretching exercise of upper and lower limbs	Computerized cognitive training (CCT)+physical exercise group: 22 Control group: 22	25 to 30 min, 5 times/week for 6 weeks	EEG	MMSE MoCA TMT‐A, B	The CCT group showed significant improvements in MMSE (*p* = 0.003) and MoCA (*p* = 0.0001) scores, attention (*p* = 0.028), and executive function (*p* = 0.0001) compared to those in the control group.	The CCT+physical exercise group showed a significant improvement in delta wave activity (*p* = 0.04) and complexity of EEG (*p* = 0.016) compared to those in the control group.
Li et al. (2019)^39^	Eight tasks of an online computerized cognitive training (CCT) program at each patient's home Control	CCT group: 80 Control group: 80	120–160 min/week, 3‐4 sessions/week for 6 months	Functional MRI	MMSE ACER AVLT STT RCFT SDMT SCWT	The CCT group showed significant improvements in MMSE score (*p* < 0.05), attention (*p* < 0.05), memory (*p* < 0.05), visuospatial configuration (*p* < 0.05), and executive function (*p* < 0.05) compared to those in the control group.	The CCT group showed significantly increased regional activity at the bilateral temporal poles, insular cortices, and hippocampus compared to those in the control group (*p* < 0.05); however, there was no significant training effects 12 months after the last training session.
Damirchi et al. (2018)^25^	Physical training (PH) Mental training (ME); special computer gaming PH + ME Control	PH group: 11 ME group: 11 PH + ME group: 13 Control group (CO): 9	PH, 30–60 min, 3 times/week for 8 weeks ME, 30–60 min, 3 times/week for 8 weeks PH + ME, an interval of 45 min between PH and ME, completed their tasks same as the first and second groups CO, none	Serum BDNF	WAIS‐Ⅲ forward digit span test WAIS‐Ⅲ digit symbol coding test SCWT	The ME group showed a significant increase in working memory compared to that in the CO group (*p* = 0.012). The ME group also showed a significant increase in working memory (*p* = 0.014) and processing speed (*p* = 0.024) compared to those in the PH group.	The ME group showed a significant increase in BDNF level compared to that in the CO group (*p* = 0.024).
Anderson‐Hanley et al. (2018)^40^	Computerized cognitive training (CCT)+physical exercise (PE) (i.e., while exergaming using a cybercycle) Game only	CCT + PE group: 91 Game only group: 15	CCT + PE: at least 20 min at least twice a week, with gradual increase in exercise duration to 45 min and frequency to at least three to five times per week until 3 months, and then maintenance of that pattern through 6 months Game only: no physical exercise	Saliva (BDNF, CRP, IL‐6, VEGF, miRNA‐9) Structural MRI	SCWT Color trials Digit span MoCA Ecological Validity Questionnaire ADAS immediate and delayed recall	The CCT + PE group showed significant improvements in executive function (*p* < 0.05) and verbal memory (*p* < 0.05) after 6 months.	Exercise dose was significantly associated with increased brain‐derived neurotropic factor level (*p* = 0.04). Exosome analyses revealed a significant positive correlation between microRNA‐9 expression and improved SCWT performance (*p* = 0.002). Greater exercise dose was positively correlated with increasing prefrontal cortex volume (*p* = 0.003). Verbal memory errors were inversely related to increasing dorsal lateral prefrontal cortex volume (*p* = 0.01).
Suo et al. (2016)^12^	Computerized cognitive training, COGPACK program (CCT) + progressive resistance training (PRT) CCT+Sham PRT+Sham Control (Sham+Sham)	CCT + PRT group: 27 CCT+Sham group: 24 PRT+Sham group: 22 Control group: 27	90 min, 2 day/week for 26 weeks	Structural MRI Functional MRI	ADAS‐Cog	The combined CCT and PRT group showed a significant improvement in global cognition compared to those in the control group (*p* < 0.05). The CCT group showed no decline in memory domain scores compared to the control group (*p* < 0.02).	The CCT + PRT group showed significantly increased cortical thickness in the posterior cingulate, related to improvement in global cognitive function (*p* = 0.03). The CCT group showed significantly enhanced functional connectivity between the hippocampus and superior frontal cortex related to improvement in memory (*p* < 0.05).
Rosen et al. (2011)^41^	Computer‐based cognitive training program (CCT) Control: listening to audio books, reading online newspapers, playing a computer game	CCT group: 6 Control group: 6	CCT: 100 min, 5 day/week for 2 months Control: 90 min, 5 days per week for 2 months	Functional MRI	Repeatable Battery for the Assessment of Neuropsychological Status, 30	The CCT group showed a significant improvement in verbal memory compared to the control group (*p* = 0.027).	The CCT group showed significantly increased hippocampal activation compared to that in the control group with no correlation with memory function (*p* value was not showed).

Abbreviations: ACER, Addenbrooke's Cognitive Examination‐Revised; ADAS‐Cog, Alzheimer's Disease Assessment Scale‐Cognitive subscale; ApoE, apolipoprotein E; AVLT, Auditory Verbal Learning Test; BDNF, brain‐derived neurotropic factor; BNT, Boston Naming Test; CRP, C‐reactive protein; CVVLT, Chinese Version of the Verbal Learning Test; EEG, electroencephalography; EXIT‐25, Executive Interview‐25; fNIRS, functional near‐infrared spectroscopy; IADL, instrumental activities of daily living; IL‐6, interleukin‐6; LMX1A, Lim homeobox transcription factor‐alpha; miRNA‐9, exosomal microRNA‐9; MMSE, Mini‐Mental State Examination; MoCA, Montreal Cognitive Assessment; MRI, magnetic resonance imaging; NSE, neuron‐specific enolase; RCFT, Rey‐Osterrieth Complex Figure Test; S100β, S100 calcium‐binding protein B; SCWT, Stroop Color‐Word Test; SDMT, Symbol Digit Modalities Test; STT‐A,B, Shape Trail Test‐A,B; TMT‐A, B, Trail Making Test‐A, B; VEGF, vascular endothelial growth factor; VFT, Verbal Fluency Test; VLT, California Verbal Learning Test; WAIS‐Ⅲ, Wechsler Adult Intelligence Scale‐Ⅲ; WMS‐Ⅲ, Wechsler Memory Scale‐Ⅲ.

### 3.2 Characteristics of the included studies

The analysis involved a total of 1,115 participants. The mean age of participants in this study was 71.1 years.　The studies were conducted in Europe (*n* = 1), Oceania (*n* = 2), the United States (*n* = 3), Asia (*n* = 9), and other regions (*n* = 1). The diagnosis of MCI was made using diagnostic criteria such as Petersen's diagnostic criteria,^1^ with neuropsychological rating scales (*n* = 7). MCI screening was performed based on established neuropsychological evaluation scales such as Montreal Cognitive Assessment and Clinical Dementia Rating (*n* = 9).

### 3.3 Biomarkers assessed in the included studies

The 16 extracted RCT studies were divided into five categories: (1) structural magnetic resonance imaging (MRI), (2) functional MRI, (3) EEG, (4) functional near‐infrared spectroscopy (fNIRS), and (5) serum and salivary biomarkers. All the studies estimated the correlations between biomarkers and cognitive functional outcomes.

### 3.4 Structural MRI (*n* = 4)

Four studies reported volumetric and cortical thickness outcomes after interventions. Suo et al.^12^ divided participants into four groups: CCT (COGPACK program) + progressive resistance training (PRT), sham CCT + PRT, CCT+sham PRT, and double sham. Both the CCT and PRT consisted of sessions lasting 90 min/day, 2 days/week, for 26 weeks. The results showed a significant increase in cortical thickness in the posterior cingulate cortex (PCC) with improvement in global function in the CCT + PRT group and attenuated decline in overall memory performance with no volumetric change in the CCT group. Twelve months after training cessation, the atrophy rates in the left hippocampus were significantly slower in the PRT + CCT group than in the double sham group.^37^ Anderson‐Hanley et al.^40^ conducted mental and physical exercises, starting with a minimum of 20 min at least twice a week and gradually increasing the exercise duration to 45 min and frequency to at least three to five times a week for 3 months and maintained that pattern through a 6‐month period. The results showed a significant improvement in executive function, and exercise dose was associated with increased gray matter volume in the prefrontal cortex (PFC) and anterior cingulate cortex (ACC). In addition, a significant improvement in memory was associated with increased gray matter volume in the dorsal lateral PFC (DLPFC). In another study, CCT performed at home using the Cogmed working memory (WM) training program revealed no structural cortical changes after the intervention; the training was conducted for 30–40 min, five times/week for 5 weeks. However, the results revealed significantly increased cortical thickness in the right frontal superior region and right paracentral region in Lim homeobox transcription factor‐alpha (LMX1A)‐AA carriers of the LMX1A polymorphism, associated with the maintenance of dopaminergic neurons and disorders, such as Parkinson's disease.^35^

### 3.5 Functional MRI (fMRI) (*n* = 7)

A task‐based MRI study revealed that participants in the CCT group, with CCT, conducted 100 min/day, 5 days per week for 2 months, showed higher hippocampal activation with improvement in verbal memory than participants in the control group.^41^ Resting‐state fMRI (rsfMRI) had shown significantly enhanced FC between the hippocampus and superior frontal cortex, with attenuation of memory decline in the CCT group after intervention with the COGPACK program.^12^ In a study involving an online CCT program at the patient's home for 120–160 min/week with 3–4 sessions per week for 6 months, rsfMRI showed significantly increased regional activity at the bilateral temporal poles, insula cortices, and hippocampus with improvement in Mini‐Mental State Examination score, attention, memory, visuospatial configuration, and executive function. However, training effects were not significant 12 months after the last training.^39^ Another follow‐up study conducted 12 months after intervention cessation by Suo et al. found significantly enhanced FC between the hippocampus and PCC, with no long‐term cognitive improvement in the CCT + PRT group.^37^ In a VR‐based cognitive training session that was conducted for 20–30 min, twice per week for 4 weeks, rsfMRI revealed significantly increased FC between the frontal and occipital regions associated with improvement in the Rey–Osterrieth Complex Figure Test (RCFT) score.^9^ A rsfMRI study on computerized crossword training for four 30‐min per week sessions for 12 weeks and subsequent booster training until 72 weeks showed significantly enhanced FC within the default mode network (DMN) and FC between the DMN and salience network (SLN) with improvement in the 11‐item Alzheimer's Disease Assessment Scale‐Cognitive total score.^29^, ^34^ Another rsfMRI study using the Remote Expressive Arts Program for 60 min, twice weekly for 12 weeks, found significantly increased activation in the right ACC and left dorsolateral superior frontal gyrus related to improved attention, memory, and global cognitive function.^33^

### 3.6 Electroencephalography (EEG) (*n* = 3)

A study using the Xbox 360 Kinect CCT program for 25–30 min, five times per week for 6 weeks, showed significantly lower delta waves and complex EEG with improvements in attention, executive function, and global cognitive function in the intervention group than in the control group.^38^ In another two studies conducted by the same research group, the VR‐based cognitive training (VRCT) group (100 min, 3 times/week for 8 weeks) showed significantly lower theta/beta ratio in the temporal and parietal regions with improvements in executive function compared with the control group.^19^, ^32^ A comparison between the VRCT and exercise groups showed significantly lower theta power in the parietal area and lower connectivity in the PFC, ACC, and temporal and parietal regions in the VRCT group than in the exercise group.^32^

### 3.7 Functional near‐infrared spectroscopy (fNIRS) (*n* = 2)

Two studies used fNIRS as a biomarker.^17^, ^36^ Compared with VR‐based physical and cognitive training (VR training: physical training for 40 min, cognitive training for 20 min, 3 times/week for 12 weeks) and combined physical and cognitive training (60 min, 3 times/week for 12 weeks), the results showed significantly decreased activation in the prefrontal areas after training in both groups and an improvement in global cognitive function, verbal memory, and instrumental activities of daily living (IADLs) in only the VR training group.^17^ Another study compared cognitive–physical dual‐task training (40 min, twice/week for 8 weeks) with only executive function training (40 min, twice/week for 8 weeks). Compared with the single cognitive task, the cognitive–physical dual‐task training showed significantly better executive process and lower prefrontal activity during cognitive testing, but no significant differences were noted in IADLs between the groups.^36^

### 3.8 Serum or salivary biomarkers (*n* = 3)

The comprehensive and complex cognitive stimulation program consisted of 120 min of computer activity, physical activity, and exchanging experiences in a conversation circle two times/week for 12 weeks. The intervention showed significantly decreased serum BDNF levels with improvement in global cognitive function but with no significant change in serum S100 calcium‐binding protein B or neuron‐specific enolase levels.^14^ Aerobic and cognitive exercises (mentioned before) significantly increased salivary BDNF, vascular endothelial growth factor (VEGF), and exosomal microRNA‐9 (miRNA‐9) expressions, with improvement in memory and executive function after intervention.^40^ In another study, participants were randomized into four groups: physical training; mental training, that is, CCT (30–60 min, 3 times/week for 8 weeks); combined training; and control. The results showed a significant increase in serum BDNF levels in the mental training group, with improvements in WM compared with that noted in the control group.^25^

## 4 DISCUSSION

We included 16 studies and reviewed the types and findings of biomarkers to evaluate the effects of CCT in older adults with MCI. The biomarkers extracted in our study included MRI, EEG, fNIRS, and serum and salivary biomarkers.

### 4.1 MRI

Among the articles extracted in our study, MRI was the most frequently used. As two of the four structural MRI studies also examined connectivity within cerebral regions, we have discussed structural and functional MRI together. CCT enhances regional activity in areas of the hippocampus related to improvement in verbal memory^41^ and in the temporal poles and insular cortices, which are related to improvement in global cognitive function, attention, memory, visuospatial configuration, and executive function.^39^ It also enhances regional activity in the ACC and superior frontal cortex, leading to improved attention, memory, and global cognitive function.^33^ Regarding the FC between brain regions, CCT enhanced the FC between the hippocampus and superior frontal cortex related to improvement in memory,^12^ between the hippocampus and PCC,^37^ between the frontal and occipital regions related to improvements in the RCFT copy task,^9^ and between the DMN and SLN with improvement in overall cognitive function.^29^ These studies have focused on structural and functional changes within major brain networks, such as the DMN, executive control network (ECN), and SLN. Furthermore, changes in these major brain networks by CCT were investigated.

The main brain regions involved in the DMN are the PCC, medial PFC, ACC, and inferior parietal lobule.^42^ In particular, the PCC, which plays a vital role in the DMN, receives input from several regions, including the hippocampus, and engages in cognitive functions such as memory, attention, and visuospatial cognition.^43^ The FC between the PCC and hippocampus is attenuated from the MCI stage due to AD.^44^ Possible reasons for the efficacy of CCT in older patients with MCI include the recovery of weakened FC between the PCC and hippocampus, enhancement of FC within the DMN, and an increase in FC between the DMN and other major brain networks, including the SLN.^29^, ^37^

The ECN is composed of the DLPFC, ventrolateral PFC, and PCC^45^ and plays crucial roles in the integration of sensory and memory information, the regulation of cognition and behavior, and the WM process with the prefrontal lobe.^46^ Imaging biomarkers in MCI include changes in specific brain regions in the ECN, primarily the precuneus, cuneiform nucleus, lingual gyrus, middle frontal gyrus, and PCC.^47^ The improvement in cognitive function, including executive function, by CCT may be related to an increase in the volume of the DLPFC, which is a component of the ECN, and the PCC, which is a component common to the ECN and DMN.^12^, ^40^

Another major brain network, i.e., the SLN, primarily comprises the insula and dorsal ACC, which participate in attention and switching between cognitive resources.^48^ The SLN is significantly affected in patients with MCI,^49^ and specific functional alterations in the SLN and interactions of the SLN with the DMN in MCI may be useful as potential imaging biomarkers for MCI.^50^ One possible reason for the efficacy of CCT in older patients with MCI is the enhanced regional activity in the insula and ACC.^29^, ^33^, ^39^

However, some studies have reported contradictory results in these contexts. For example, studies have reported no structural change after WM training^35^ or enhanced FC in the neural network after CCT involving non‐crossword puzzles.^29^ Another study demonstrated that FC changes in the PCC negatively correlated with improvement in delayed memory.^33^ One possible reason for these results is the diversity of MCI. Cognitive function and MRI findings following CCT may differ according to the cause of MCI, such as AD or Lewy body disease, and in the early and late stages of MCI.^29^ The second reason may be associated with a compensatory mechanism. FC within the DMN increases as a compensatory mechanism to maintain cognitive function in MCI due to AD.^51^ CCT can help in recovering the increased FC within the brain network after improvement in cognitive function. Other potential reasons may be related to the dose and duration of CCT. A greater exercise dose is positively correlated with increased PFC and ACC volumes.^40^ A certain dose and duration of CCT may be required for certain changes to be evident using MRI. Furthermore, analysis of genes related to the maintenance of neural function revealed that MRI findings after training differed depending on gene polymorphisms.^35^ Thus, investigating the genetic polymorphisms associated with neural function may be important for predicting the effects of training.

### 4.2 EEG

Previous EEG studies in patients with MCI revealed consistent neural alterations compared to those in healthy adults, involving decreased alpha and beta rhythm activities, increased delta and theta power band activities,^52^ and decreased EEG complexity.^53^ Theta waves increase because of the slower axonal conduction time in subcortical areas.^54^ Slower and less complex EEG patterns are caused by damage to cholinergic neurons in the lateral capsular and perisylvian pathways, which play important roles in cortical activity.^53^, ^55^ In our study, EEG was reported to significantly decrease theta rhythm activity in the temporal and parietal regions after CCT.^19^, ^32^ These studies suggest that CCT can help recover damaged cholinergic neurons in extensive brain regions, leading to improvements in attention, executive function, and global cognitive function.^19^, ^32^, ^38^

### 4.3 fNIRS

fNIRS can measure alterations in the hemoglobin concentration on the cortical surface in response to neuronal activity.^56^ Hemodynamic data revealed significantly decreased activation in the prefrontal areas after training in VR‐trained participants,^17^ which is consistent with the result of a previous study.^57^ In MCI, compensatory functions are activated, and the activity of a wide range of neural networks increases to maintain brain function.^58^ When cognitive training stimulates neuronal plasticity, the burden on neural activity during task is reduced, resulting in less active neural networks.^57^, ^59^, ^60^ Changes in prefrontal activation on fNIRS after the intervention may be a biomarker for estimating the effects of CCT. In the studies analyzed herein, 16‐ or 8‐channel fNIRS was used to assess hemodynamic data in the PFC. fNIRS using more multichannel, for example, 54‐channel fNIRS, may be required to evaluate activation in all brain regions.

### 4.4 Blood or salivary biomarkers

BDNF, VEGF, and miRNAs are biomarkers that are measurable using blood or saliva. Our results indicate that post‐intervention BDNF levels increased or decreased, with contradictory results. BDNF belongs to the family of neurotrophic proteins, is important for the normal development of the central and peripheral nervous systems, and plays a prominent role in the development, survival, and function of neurons.^61^ BDNF is a central biomarker of neuroplasticity, and several studies have shown increased BDNF levels after physical or cognitive training.^25^, ^62^ In contrast, Quialheiro et al.^14^ reported that increased BDNF levels, which reflect a compensatory mechanism in MCI, decreased after interventions. However, it may be difficult to evaluate the intervention effects at the BDNF level in older adults with MCI.

Growth factors of the VEGF family play an important role in the protection and recovery from ischemia by regulating angiogenesis.^63^ miRNAs in circulating exosomes play a key role in neurodegenerative diseases, such as AD, affecting several processes involved in disease pathology.^64^ VEGF and miRNA levels decrease in neurodegenerative diseases and may increase postexercise,^65^, ^66^ making them potential biomarkers to investigate intervention effects. However, few studies have investigated these as biomarkers of intervention effects in older adults with MCI, and further studies are required.

### 4.5 Drop‐out rates in the included studies

All studies extracted in this study mentioned drop out. Drop‐out rates varied from 0%^29^, ^36^ to 87%^40^ in the intervention groups in this study, possibly influenced by cognitive function^39^ and motivation^67^ in the subjects, and duration of the intervention^67^ as has been reported by other authors.^39^, ^67^ This finding underscores the importance of recognizing that CCT programs may not necessarily allow participants to continue training without difficulty. Thus, CCT programs that consider the interests, cognitive abilities, and physical function of older adults with MCI are warranted.

### 4.6 Combined intervention and virtual reality‐based cognitive training

In our study, 8 of the 16 studies included were conducted using combined CCT and physical exercise. A meta‐analysis of RCTs demonstrated that the combination of CCT and physical exercise had positive effects on cognitive function, including executive function and attention, compared with the effects of CCT alone.^68^ In our study, CCT showed significantly lesser atrophy in the PCC in the physical exercise group than in the CCT alone group,^12^ and MRI performed 12 months after training cessation showed significantly enhanced FC between the hippocampus and PCC than in the CCT alone group.^37^ Given the results of these studies using biomarkers, combined interventions may be more effective and longer‐lasting than CCT alone.

However, physical exercise alone causes structural and functional changes in the brain^69^; therefore, changes in cognitive function and biomarkers with CCT alone should be assessed with caution. In contrast, CCT was found to improve cognitive function but not lead to improvement in instrumental IADLs.^36^ In contrast, VR‐based cognitive training improved not only executive function but also IADLs.^17^ The advantage of VR training is that it improves visuospatial processing through repeated presentation of real‐world dynamic, multisensory, and interactive environments.^9^ VR‐based training is thought to enhance the parietal lobe function and FC between the frontal and occipital networks, resulting in improved IADLs.^9^ The intervention program by Liao et al., incorporated elements that actively involved participants in community scenarios, asking them to perform tasks such as using transportation, grocery shopping, and meal preparation, directly tapping into IADL.^17^ Additionally, cognitive leisure activities and day service use can be associated with slower cognitive decline.^70^, ^71^ Thus, it is important to develop VR‐based training programs that can be applied in home settings, functioning as cognitive leisure activities and potentially contributing to long‐term cognitive benefits.

### 4.7 Limitations

This study has some limitations. First, the quality of each study was not determined, potentially influencing the results. While in line with the methods for a scoping review, this limitation indicates the need for future systematic reviews to address these issues. Second, the studies included a diverse range of cognitive training techniques, biomarkers, and patient populations, including varying causes of MCI, such as AD or Lewy body disease, and early and late stages of MCI. Therefore, these variations should be considered when interpreting the results. Third, half of the included studies performed CCT in combination with physical exercise, and the effects of physical exercise on cognitive function and biomarkers were only partially accounted for. Fourth, we only included articles published in English with the full text available, which could have led to the exclusion of some important articles in this review. Finally, we performed a bibliographic search of only three databases and therefore might have missed some important articles.

### 4.8 Direction for future intervention efforts

First, none of the articles included in our study described the causes of MCI. Cognitive impairment^72^ and behavioral and psychological symptoms^73^ that tend to appear in older individuals with MCI differ depending on the specific underlying disease. Therefore, effective intervention methods and biomarkers may also differ. Future studies should consider intervention methods and biomarkers for each cause of MCI and each genetic polymorphism associated with neural function. Second, the number of study participants was small. Therefore, it is necessary to confirm the usefulness of biomarkers for evaluating intervention effects in a larger number of participants. Third, this study did not clearly specify whether CCT could be continued after the intervention was completed, indicating the need for further research. Finally, only two studies^37^, ^39^ attempted to assess the duration of the effect of using biomarkers 1 year after the intervention ended. Biomarkers also require long‐term observation.

## 5 CONCLUSION

We reviewed the effects of CCT on biomarker outcomes in older adults with MCI. The biomarkers extracted in our study were MRI, EEG, fNIRS, and serum or salivary biomarkers, including BDNF. We found that CCT causes functional and structural changes in extensive brain regions in older adults with MCI through neuroimaging, including MRI, and improves cognitive function. The causes of MCI are diverse, and the biomarkers measured may differ depending on the cause. Future studies should consider intervention methods and biomarkers for each cause of MCI.

## AUTHOR CONTRIBUTIONS

**Hiroshi Hayashi**: Conceptualization; data curation; methodology; writing—original draft; writing—review and editing. **Toshimasa Sone**: Data curation; formal analysis; methodology; writing—review and editing. **Kazuaki Iokawa**: Data curation; writing—review and editing. **Koshi Sumigawa**: Visualization; writing—review and editing. **Takaaki Fujita**: Validation; writing—review and editing. **Hironori Kawamata**: Validation; writing—review and editing. **Akihiko Asao**: Validation; writing—review and editing. **Iori Kawasaki**: Validation; writing—review and editing. **Maki Ogasawara**: Visualization; writing—review and editing. **Shinobu Kawakatsu**: Data curation; supervision; validation; writing—review and editing.

## CONFLICT OF INTEREST STATEMENT

The authors declare no conflict of interest.

## TRANSPARENCY STATEMENT

The lead author Hiroshi Hayashi affirms that this manuscript is an honest, accurate, and transparent account of the study being reported; that no important aspects of the study have been omitted; and that any discrepancies from the study as planned (and, if relevant, registered) have been explained.

## Data Availability

All data generated or analyzed during this study are included in this published article.
